# Autologous haematopoietic stem cell transplantation for multiple sclerosis: a position paper and registry outline

**DOI:** 10.1177/17562864231180730

**Published:** 2023-09-28

**Authors:** Antonios Bayas, Achim Berthele, Norbert Blank, Peter Dreger, Simon Faissner, Manuel A. Friese, Lisa-Ann Gerdes, Oliver Martin Grauer, Vivien Häussler, Christoph Heesen, Dietlinde Janson, Mirjam Korporal-Kuhnke, Markus Kowarik, Nikolaus Kröger, Jan D. Lünemann, Roland Martin, Uwe Meier, Sven Meuth, Paolo Muraro, Michael Platten, Lucas Schirmer, Klarissa Hanja Stürner, Jan Patrick Stellmann, Christof Scheid, Florian Then Bergh, Clemens Warnke, Brigitte Wildemann, Tjalf Ziemssen

**Affiliations:** Department of Neurology and Clinical Neurophysiology, Faculty of Medicine, University of Augsburg, Augsburg; Department of Neurology, School of Medicine, Technical University of Munich, Munich; Rheumatology Section, Interdisciplinary Centre for Chronic Inflammatory Diseases, Heidelberg University Hospital, Heidelberg; Spokesman German Working Group for Haematopoietic Stem Cell Transplantation and Cellular Therapy e.V., Heidelberg University Hospital, Heidelberg; Department of Neurology, University Hospital of Ruhr-University Bochum, St. Josef-Hospital, Bochum; Institute of Neuroimmunology and Multiple Sclerosis (INIMS) and Department of Neurology, University Medical Center Hamburg-Eppendorf; Institut für Klinische Neuroimmunologie am Klinikum der Ludwig-Maximilians-Universität München, München; Department of Neurology with Institute for Translational Neurology, University Hospital Münster, Münster; Institute of Neuroimmunology and Multiple Sclerosis (INIMS) and Department of Neurology, University Medical Center Hamburg-Eppendorf, Hamburg; Institute of Neuroimmunology and Multiple Sclerosis (INIMS) and Department of Neurology University Medical Center Hamburg-Eppendorf; Clinical and Rehabilitative MS Research, Institute for Neuroimmunology and Multiple Sclerosis (INIMS), University Medical Center Hamburg-Eppendorf (UKE), Martinistrasse 52, D-20246 Hamburg, Germany; Clinic for Stem Cell Transplantation, University Medical Center Hamburg-Eppendorf, Hamburg; AG Neuroimmunology, Neurological Clinic, Heidelberg University Hospital, Heidelberg; Department of Neurology & Stroke, and Hertie Institute for Clinical Brain Research, Eberhard-Karls University of Tübingen, Tübingen, Kröger; Clinic for Stem Cell Transplantation, University Medical Center Hamburg-Eppendorf, Hamburg; Department of Neurology with Institute of Translational Neurology, University Hospital Münster, Münster; Institute of Experimental Neurology, University Hospital Zurich, Zurich, Switzerland; Chairman of the Professional Association of German Neurologists, Neurocentrum Grevenbroich, Grevenbroich; Medical Faculty, Department of Neurology, University Hospital Düsseldorf, Düsseldorf; Department of Brain Sciences, Imperial College London, London, UK; Department of Neurology, Mannheim Center for Translational Neuroscience, Medical Faculty Mannheim, Heidelberg University, Heidelberg; Department of Neurology, Mannheim Center for Translational Neuroscience, Medical Faculty Mannheim, Heidelberg University, Heidelberg; Department of Neurology, Christian-Albrechts-Universität Kiel, Kiel; Centre de Résonance Magnétique Biologique et Médicale, Aix-Marseille Université, Marseille; Clinic I for Internal Medicine, University Hospital Cologne, Cologne; Clinic and Polyclinic for Neurology, University of Leipzig, Leipzig; University of Cologne, Faculty of Medicine and University Hospital Cologne, Clinic and Polyclinic of Neurology, Cologne; AG Neuroimmunology, Neurological Clinic, Heidelberg University Hospital, Heidelberg; Center of Clinical Neuroscience, Department of Neurology, University Clinic Carl Gustav Carus Dresden, Technische Universität Dresden

**Keywords:** Autologous haematopoietic stem cell transplantation (AHSCT), multiple sclerosis, registry study, treatment recommendation

## Abstract

**Background:**

While substantial progress has been made in the development of disease-modifying medications for multiple sclerosis (MS), a high percentage of treated patients still show progression and persistent inflammatory activity. Autologous haematopoietic stem cell transplantation (AHSCT) aims at eliminating a pathogenic immune repertoire through intense short-term immunosuppression that enables subsequent regeneration of a new and healthy immune system to re-establish immune tolerance for a long period of time. A number of mostly open-label, uncontrolled studies conducted over the past 20 years collected about 4000 cases. They uniformly reported high efficacy of AHSCT in controlling MS inflammatory disease activity, more markedly beneficial in relapsing-remitting MS. Immunological studies provided evidence for qualitative immune resetting following AHSCT. These data and improved safety profiles of transplantation procedures spurred interest in using AHSCT as a treatment option for MS.

**Objective:**

To develop expert consensus recommendations on AHSCT in Germany and outline a registry study project.

**Methods:**

An open call among MS neurologists as well as among experts in stem cell transplantation in Germany started in December 2021 to join a series of virtual meetings.

**Results:**

We provide a consensus-based opinion paper authored by 25 experts on the up-to-date optimal use of AHSCT in managing MS based on the Swiss criteria. Current data indicate that patients who are most likely to benefit from AHSCT have relapsing-remitting MS and are young, ambulatory and have high disease activity. Treatment data with AHSCT will be collected within the German REgistry Cohort of autologous haematopoietic stem CeLl trAnsplantation In MS (RECLAIM).

**Conclusion:**

Further clinical trials, including registry-based analyses, are urgently needed to better define the patient characteristics, efficacy and safety profile of AHSCT compared with other high-efficacy therapies and to optimally position it as a treatment option in different MS disease stages.

## Plain language summary

Autologous haematopoietic stem cell transplantation for multiple sclerosis

Substantial progress has been made in the development of disease-modifying medications for multiple sclerosis (MS) during the last 20 years. However, in a relevant percentage of patients, the disease cannot completely be contained. Autologous haematopoietic stem cell transplantation (AHSCT) enables rebuilding of a new and healthy immune system and to potentially stop the autoimmune disease process for a long time. A number of studies documenting 4000 cases cumulatively over the past 20 years reported high efficacy of AHSCT in controlling MS inflammatory disease activity. These data and improved safety profiles of the treatment procedures spurred interest in using AHSCT as a treatment option for MS.

An open call among MS neurologists as well as among experts in stem cell transplantation in Germany started in December 2021 to join a series of video calls to develop recommendations and outline a registry study project.

We provide a consensus-based opinion paper authored by 25 experts on the up-to-date optimal use of AHSCT in managing MS. Current data indicate that patients are most likely to benefit from AHSCT if they are young, ambulatory, with high disease activity, that is, relapses or new magnetic resonance imaging (MRI) lesions. Treatment data with AHSCT will be collected within the German REgistry Cohort of autoLogous haematopoietic stem cell transplantation MS (RECLAIM).

Further clinical trials including registry-based analyses and systematic follow-up are urgently needed to better define the optimal patient characteristics as well as the efficacy and safety profile of AHSCT compared with other high-efficacy therapies. These will help to position AHSCT as a treatment option in different MS disease stages.

## Introduction

Autologous haematopoietic stem cell transplantation (AHSCT) is becoming increasingly important internationally in treating multiple sclerosis (MS).^1,2^ While only two randomised controlled trials (RCT) have been completed so far, which had included 21 and 110 patients, respectively,^3,4^ several retrospective data analyses, meta-analyses and systematic reviews have gathered data from more than 4000 AHSCTs documented in publications worldwide.^5–8^ However, many questions about using AHSCT in MS are still unsolved. The three ongoing RCT studies, STAR-MS in the United Kingdom, BEAT-MS in the United States and RAM-MS in Norway,^9^ together with the upcoming NET-MS study in Italy, aim to confirm effects in highly active relapsing-remitting MS in comparison with high-efficacy treatments such as B cell depleting and other monoclonal antibodies. Data should be available within 3–4 years. Being aware that MS is a progressive disease from disease onset,^10^ the possibly most relevant questions are which is the optimal window of opportunity for AHSCT as an alternative to standard therapy and what extent of clinical progression in relation to disease duration and age justifies AHSCT as a rescue therapy.^11^ Other major issues are the choice of the conditioning regimen (intensity of lymphoablation and extent of myeloablation required) and establishing the long-term safety in relation to the conditioning, in respect of the risks of malignancies and secondary autoimmune diseases.

In Germany, AHSCT for MS in general is not paid for by insurance companies and hence is only performed on an individual basis and expert opinion. Therefore, coverage needs to be negotiated from case-to-case and after rejection of compensation from insurance companies, patients need to pay on their own. Some try to reach out for a social court verdict. Neurologists have been mostly reluctant, which has led to only 57 patients being documented in the European Society for Blood and Marrow Transplantation (EBMT) database of 1.721 cases between 1995 and 2021.^9^ In addition, insurance companies in most cases do not cover treatment costs. Therefore, a relevant number of persons with MS (pwMS) opted for treatment to be performed in other countries in the past. Knowledge about the benefits and risks of AHSCT among German pwMS is sparse. To overcome the treatment and knowledge gap in Germany, MS neurologists and stem cell transplantation experts have joined to develop recommendations for AHSCT treatment in MS and strategies to gather treatment experience.

This article represents recommendations developed as an overarching expert consensus by a task force on stem cell transplantation in MS established under the umbrella of the Clinical Competence Network Multiple Sclerosis (KKNMS), the German Working Group for Haematopoietic Stem Cell Transplantation and Cellular Therapy e.V. (DAG-HSZT), and the German MS Self-Help Association (DMSG). They are intended to help identify patients who are most suitable for AHSCT, to facilitate the coverage of costs by the health insurances, and to carry out and follow up the transplantations according to a common standard and document them in the German MS registry and the German Registry for Stem Cell Transplantation (DRST). The goal is to systematically document all experience with AHSCT in MS in Germany, including cases beyond the consensus criteria mentioned below, to specify clear indication criteria based on long-term data.

### Criteria for the use of AHSCT

We will distinguish between narrower and broader criteria aligned with the Swiss standard for AHSCT in MS.^12^ The narrower criteria correspond to the core criteria of EBMT^9^ and the criteria of the American MS Society (National MS Society, NMSS)^13^ based on recommendations of the American Society for Blood and Marrow Transplantation (ASBMT).^14^

Although AHSCT is probably the most effective immunotherapy for MS and can reset the immune system, it is, at least based on our current knowledge, not a regenerative therapy. Current evidence indicates that young persons with a short disease duration course of MS, and high inflammatory activity on magnetic resonance imaging (MRI) (new T2 lesions and contrast agent lesions) and a high relapse frequency benefit most from this approach. Data are not conclusive in progressive disease courses and in the absence of MRI activity. For advanced disease stages with a long duration, older age and greater impairment, data argue against a benefit that would justify the risks of transplantation. Recent inflammatory activity on MRI is included in all current criteria. First introduced in a short section on evidence, the criteria are discussed individually and then summarised in tabular form for relapsing and secondary progressive MS (SPMS). Primary progressive MS (PPMS) is discussed separately at the end.

#### Disease course

AHSCT is primarily used to treat relapsing-remitting MS. The data on SPMS suggest a significantly lower efficacy. In the meta-analysis by Sormani *et al.*,^15^ relapsing and non-relapsing patients were compared. Here, a clear difference was found in favour of RRMS. Treatment-related mortality (TRM) was also significantly lower in RRMS. In the meta-analysis by Zhang and Liu,^5^ RRMS showed 81% progression-free survival (PFS), SPMS 78% and PPMS 60%. In a long-term observed cohort of 281 evaluable patients, with median follow-up of 6.6 years, Muraro *et al.*^16^ reported significantly worse PFS in progressive *versus* relapsing form of MS [hazard ratio (HR), 2.33; 95% confidence interval (CI), 1.27–4.28].

In the real-world case series by Burt *et al.*,^17^ PFS at 4 years was 95% in RRMS and 66% in SPMS. The Italian experience in *n* = 210 pwMS from 20 Italian centres was reported by Boffa *et al.*^18^ In all, 86 patients were diagnosed SPMS and 2 patients PPMS. PFS was 85.5% in RRMS at 5 years compared with 71% in progressive group. Another analysis of the subgroup of SPMS with *n* = 79 from the Italian cohort applied a matching analysis with established MS treatments and confirmed a longer time to disability progression in active SPMS after AHSCT (HR = 0.50; 95% CI = 0.31–0.81; *p* = 0.005).^11^ In the series by Nicholas *et al.*,^19^ no differences in the effect on PFS between RRMS and progressive MS could be detected. Neither demographics nor progression nor type of prior therapy were predictive.^11,17^

*Recommendation*: Active RRMS despite highly effective immunotherapy is the key indication for AHSCT. In younger patients with active SPMS and a conversion from RRMS no longer than within the preceding 3 years without severe impairment (Expanded Disabilty Status Scale Score, EDSS ⩽ 6.5), the use of AHSCT can be considered.

#### Age

Beyond the age of 50, AHSCT, like all other highly effective MS immunotherapies, should be discussed critically, though very few data on the effect of age on AHSCT are available.^20^ In the meta-analysis by Sormani *et al.*,^15^ patients > and < 36 years were compared. Here, no convincing difference in therapeutic effect was found. Age did not affect mortality. The long-term follow-up case series by Muraro *et al.*,^16^ however, reported age as one significant variable affecting PFS comparing ages 18–31, 32–37, 38–44 and beyond 45.

*Recommendation*: A lower regenerative capacity of the nervous system, ageing of the immune and nervous systems, comorbidities, and the risk of other comorbidities argue against AHSCT in pwMS beyond 50 years. In older age, a short disease duration and high inflammatory activity are a pre-requisite to consider AHSCT.

#### Impairment and progression of impairment

In the meta-analysis by Sormani *et al.*,^15^ patients with EDSS > 5.5 and ⩽ 5.5 were compared. Descriptively, there was an advantage for less affected patients, but this was not significant. However, TRM was significantly higher in EDSS > 5.5. In the real-world case series by Burt *et al.*,^17^ all groups (EDSS 2–4, EDSS 4.5–6, EDSS 6.5, EDSS 7–8) benefitted from AHSCT. Muraro *et al.*,^1^ formulates that patients with an EDSS ⩾ 7 are at high risk of treatment failure. Mariottini *et al.*^21^ recently reported a comparison of AHSCT (*n* = 31) and cyclophosphamide (CYC) therapy (monthly in first year, then every 2 months in second year, *n* = 62) in SPMS (mean 40 years, 13 years MS, EDSS 6.0). After 5 years, PFS was very similar between AHSCT (70%) and CYC (81%) treated patients, while AHSCT significantly suppressed more pronounced relapse activity. In an earlier case series of *n* = 26 patients with SPMS, Mariottini *et al.*^22^ found PFS for 42% after 5 and 30% after 10 years. Recent disability accrual has a favourable prospect over impairments that exist already for many years.

*Recommendation*: A free walking distance of at least 100 m with assistance (EDSS 6.0) is generally considered the upper limit of mobility impairment for AHSCT treatment, based on the idea that the degeneration in the nervous system should not be too advanced. In individual cases, AHSCT can be considered beyond EDSS 6.0 if the patient is very young, has a short, highly aggressive course and has a high level of inflammatory activity. In the same way, a patient who is currently only mildly affected can be treated if the inflammatory activity (many relapses, high MRI activity) and neurological impairment are high during relapses. The dynamics of progression must also be taken into account. Thus, according to the EBMT criteria based on Menon *et al.*,^23^ an EDSS 6.0 after a maximum of 5 years of disease progression or an EDSS 6.0 before age 40 are also accepted as criteria.

#### Disease duration

Detailed analyses are not available. However, the effects of disease duration often closely resemble those of age.

*Recommendation*: In general, based on the available studies, MS should not be present for longer than 10 years (first certain manifestation, not time of diagnosis), which also conforms to the idea that degeneration plays an increasing role after a longer disease course. Here, too, differentiation must be made for individual cases. If MS starts in childhood or adolescence, AHSCT can also be considered after 15 years with MS. In older patients with such a long disease period, AHSCT treatment should only be considered in cases with very high inflammatory activity.

#### Relapses

To our knowledge, data comparing the impact of high and low relapse rates before transplantation are not available.

*Recommendation*: In principle, relapse activity should be present in the period before transplantation. Given the heterogeneity of relapses and the uncertainty in diagnosing relapses, ideally, only relapses with EDSS-relevant changes should be evaluated. However, in highly active disease courses, differentiation between relapse and progression can be difficult. In case of an uncertain relapse classification, other criteria become more important for determining the indication: MRI activity, age and disease duration. In the criteria according to Menon *et al.*,^22^ an SPMS conversion since less than 3 years is mentioned. Here, however, a distinction from PPMS with superimposed relapses remains blurred.

#### MRI activity

In the real-world case series by Burt *et al.*,^17^ SPMS patients with Gd+ in the year before AHSCT were more likely to benefit. Burman *et al.*^24^ and Mancardi *et al.*,^25^ reported similar evidence.

*Recommendation*: Basically, MRI activity, that is, new contrast enhancement(s) or new/size-progressive T2 lesions in the last year, is a pre-requisite for AHSCT, although there is no international consensus on the necessary number of lesions. However, highly effective therapy can potentially lead to suppression of MRI activity in the presence of clear clinical progression. Whether this progression is neurodegenerative or an expression of diffuse inflammation not showing up on MRI is unclear. There is no doubt that contrast enhancement and T2 lesions represent only parts of the MRI inflammatory activity in MS. Therefore, affected persons without MRI activity are also suitable in individual cases, provided they are young, have a short disease duration and show considerable progression dynamics.

#### Previous immunotherapy

No data are available specifically analysing AHSCT outcomes after documented treatment failure of highly or moderately effective MS immunotherapies; however, many patients, who had received AHSCT in the past had already failed one or several approved therapies before being offered AHSCT. Indeed, one long-term observational cohort study documented the association of a higher number of previous immunotherapies with worse PFS.^16^ This observation led these authors and other experts to recommend against multiple treatment failures as a pre-requisite for consideration of AHSCT and that failure of no more than two prior disease modifying treatments (DMTs) would be preferred.^1^

*Recommendation*: In principle, treatment failure with one highly potent medication (ocrelizumab, ofatumumab, rituximab, natalizumab, alemtuzumab or a similar active substance) is required before selecting AHSCT, also in light of negotiating cost coverage with health insurance companies. In cases of aggressive disease and marked disease progression under an initial medication, AHSCT should already be considered in order not to miss the most favourable time of opportunity for this treatment. In individuals with clearly aggressive disease, AHSCT might be justified even as a first-line treatment on a case-by-case basis.

Based upon group consensus, the Task force proposes the following core and extended criteria adapting mostly the Swiss standard.^12^ The criteria listed below are not definite predictors for a response to AHSCT but are intended to describe a corridor for its use. Therefore, criteria have to be examined individually and will not necessarily be entirely fulfilled in each case. Currently, all AHSCT cases continue to be individual attempts, in German ‘Heilversuch’ to slow down the disease activity and progression (Table 1).

**Table 1.** Recommendations.

Parameter	Core criteria	Extended criteria
Age (years)	18–45	46–55
EDSS	3.0–6.0^a^	⩽ 6.5
Duration of illness (years)	⩽10	⩽15
Disease course	RRMS or SPMS with progression for ⩽ 2 years	RRMS or SPMS with progression for ⩽ 5 years
Clinical activity in the last 12–24 months	Within 12 months before: ⩾ 1 relapse with EDSS increase^b^ or ⩾ 2 relapses with or without EDSS increase^b^	Within 24 months before: ⩾ 1 relapse with or without EDSS increase^b^
Clinical progression in the last 12–24 months	Within 12 months before: Increase in EDSS^b^	Within 24 months before: Increase in EDSS^b^ or increase in other scores (MSFC) by ⩾ 20%.
MRI activity in the last 12–24 months^b^	Within 12 months before: ⩾ 1 Gd^+^ Lesion or ⩾ 1 new or enlarged T2 hyperintense lesions ⩾ 3 mm	In the last 24 months: ⩾ 1 Gd^+^ Lesion or ⩾ 1 new or enlarged T2 hyperintense lesions ⩾ 3 mm
Therapy failure	Failure of ⩾ 1 highly active substance (ocrelizumab, ofatumumab, rituximab, natalizumab, alemtuzumab or similar active)	Failure of at least 1 MS therapy

EDSS, Expanded Disbailyyt Status Scale Score; MRI, magnetic resonance imaging; MSFC, Multiple Sclerosis Functional Composite; RRMS, relapsing-remitting MS; SPMS, secondary progressive MS.

a In the case of progression during relapse, higher EDSS values can be present that justify an AHSCT, just as values < 3.0 can be present in a relapse-free interval.

b Increase in EDSS: 1 point for patients with EDSS < 5.5, 0.5 points for EDSS ⩾ 5.5.

#### Use in primary progressive MS (PPMS)

There is no specific study on PPMS, although PPMS data are available in cohorts with progressive patients. Most immunotherapies do not work convincingly in PPMS; hence AHSCT should be considered with great caution. The prototypic PPMS patient, more likely to be male, around 50 years of age, often very slowly insidious in onset, with a leading spinal course and little inflammatory activity, does not appear to be a good candidate for AHSCT. On the other hand, primary progressive inflammatory disease courses are also present in young people. Combined with the pathophysiological concept that MS is basically a progressive disease with varying degrees of focal inflammatory activity, AHSCT should also be considered in individual cases of PPMS. Ideally, these patients fulfil activity criteria through MRI activity and imposed relapses. Without MRI activity, PPMS could only be considered in cases with an aggressive course,^23^ that is, EDSS 6.0 after 5 years or EDSS 6.0 before age 40 and with enhanced consideration of the benefit *versus* risk balance.

#### Major treatment risks

A major concern of neurologists is mortality due to AHSCT which has been about 7% in the earliest treatment times from 1995 to 2000.^1^ However, TRM has substantially declined to 1% in the meta-analysis by Zhang and Liu.^5^ Looking at the most recent cases from 2017 to 2021, mortality rate was 0.2% in EBMT data^26^ as well as also 0.2% in the Burt real-world cohort.^17^ Other single-centre or regional case series reported higher rates and current estimates of risk of TRM is ~1%.^1^ In addition, secondary autoimmune diseases have been reported in about 5% and secondary cancer in about 3%. These data need to be considered with caution as long-term follow-up is limited.

### Practical management guidance of AHSCT in MS

A strong interaction of a neurological centre with profound MS expertise and a committed and experienced stem cell transplantation unit or clinic is crucial for a scientifically sound and consistent patient selection as well as for optimal treatment and follow-up including low-barrier clinical visits and any necessary readmissions. As expertise is correlated with the number of procedures performed, concentrating the work in a limited number of centres in each country is preferable.

#### Therapy decision

Decision of a neurological stem cell board (2 neuroimmunologists or MS experts, 1 haematologist stem cell transplanter, 1 neuroradiologist) is based on clinical information and MRI presentation. Written documentation of the decision is maintained in the hospital information system. To assure optimal assessment and counselling of patients, it is advisable to establish an interdisciplinary collaboration at the respective centre with fertility experts, pneumonologist, cardiologist, neuropsychologist/psychiatrist and often also gastroenterologist, and infectious disease experts.

#### Informed consent

Comprehensive patient information in oral and written form, ideally discussed in the presence of relatives and in several sessions, must address not only the opportunities but also the risks, especially the mortality risk, the risk to fertility, and the long-term risks of cancer and secondary autoimmune diseases.

#### Distance to previous therapies

General rules cannot be formulated. In view of an aggressive disease course, a critical assessment of each individual case must determine if AHSCT is justified under therapy that is still immunologically effective.

#### Preparatory investigations

Electrocardiogram (ECG); echocardiogram; chest X-ray; pulmonary function test; abdominal sonography; ear, nose and throat (ENT) and dental presentation; and negative pregnancy test are the preparatory investigations.

#### Screenings before AHSCT

Screening for CMV, EBV, VZV, HSV1 + 2, HIV, hepatitis viruses and toxoplasmosis in all patients, further infection screening as f.e. tuberculosis depending on geographical location and anamnesis are performed.

#### Mobilisation

Mobilisation is performed with 2 g/m^2^ CYC and G-CSF 10 µg/kg body weight until apheresis. Leukapheresis and cryopreservation are performed according to local standards. Cryopreservation of 3–8 × 10e6 CD34^+^ cells/kg is recommended. An additional 2.5 × 10e6 CD34^+^ cells/kg can be collected as a backup. At least 3 × 10e6 CD34^+^ cells/kg body weight are required for reinfusion. If the target cell count cannot be collected, plerixafor, a partial CXCR4 agonist, can be supplemented or a second treatment with G-CSF 10 µg/kg body weight once daily, starting 3 weeks after the first phase, combined with prednisone 1 mg/kg/body weight/day p.o. to prevent triggering of relapse. Ex vivo CD34^+^ cell selection or T cell depletion of the graft will not be performed. Once the graft is released by the professional cell manufacturer, conditioning and AHSCT can take place.

#### Conditioning

In the meta-analysis by Sormani *et al.*,^15^ high-medium and low-intensity regimens were compared. Here, only a descriptive difference in favour of high-intensity therapy was found. The conditioning regimen did not affect mortality. In the meta-analysis by Zhang and Liu,^5^ there was no clear advantage of high-intensity regimens on progression (PFS: medium intensity 73%, low intensity 85% and high-intensity 58%). However, mortality was significantly higher in high-intensity regimens (0% in low *versus* 6% in high). Analysis of EBMT data on different conditioning regimens is still pending but for conditioning both CYC and ATG (anti-thymocyte globulin) or ALG (anti-T lymphocyte globulin) or BEAM (BCNU, etoposide, cytarabine, melphalan) with ATG were mostly used with promising outcomes and manageable toxicities^9^ with a more suitable safety profile for CYC and ATG according to the real-world case series by Burt *et al.*^17^

*Recommendation*: The conditioning regimen should preferably use 200 mg/kg body weight CYC (4 × 50 mg/kg body weight). According to the safety and warning statements in the product information, treatment with CYC can lead to germline damage (oocytes and sperm). Therefore, fertile female patients should be advised about egg freezing prior to treatment, and male patients should be advised about sperm preservation.

As an alternative to CYC, the BEAM–ATG protocol can be used. To prevent engraftment of residual or reinfused autoreactive T cells, especially in case of an unselected AHSC graft, ATG (Thymoglobulin®) at a cumulative dose of 5–7.5 mg/kg body weight or ALG (Neovii®) at a cumulative dose of 60 mg/kg prior to AHSCT^17^ should be added. In addition, ATG might also support proper immune reconstitution by the induction of regulatory T cells.^27^ Premedication to avoid allergic reactions, infusion and concomitant medication are performed according to local protocols.

ATG/ATLG also requires prophylactic medication according to local standards.

#### Transplantation, prophylaxis and monitoring after infusion

Supportive care during and post-transplant should follow international and local guidelines.

After reinfusion, patients remain in hospital until engraftment is confirmed according to site guidelines. After reinfusion, the following measures are useful until discharge: daily blood count, liver, kidney and coagulation function every other day; G-CSF (5 µg/kg bw) can be administered if neutropenia persists.

All patients should remain under the direct routine care of the transplant programme specialist for at least 100 days post-transplant, or longer if needed, until clinically stable. After discharge, all AHSCT patients are monitored weekly as outpatients for up to 1 month after transplantation. Prophylaxis for herpes simplex virus reactivation includes, for example, oral acyclovir for up to 90 days and a T-helper cell count of 200/µl. Prophylaxis against *Pneumocystis jirovecii* infections with sulfamethoxazole/trimethoprim can be given for 6 months. In case of intolerance, atovaquone or dapsone can be administered alternatively, or a monthly pentamidine inhalation can be given.

All patients are monitored weekly for CMV and EBV by PCR in peripheral blood until day 30 and at every visit thereafter. Possible reactivations are monitored, and preventive treatment is given according to site guidelines. Additional routine safety visits, as recommended by the on-site haematologist/transplant specialist, are conducted according to local protocols (Table 2).

**Table 2.** Safety monitoring.

Time	Blood count, CRP, creatinine, bilirubin, transaminases, LDH	Immune status and IgG	CMV/EBV PCR
Week 1–4 after discharge	x		x
Week 4 after discharge	x	x	x
Monthly until 6 months after Tx	x	x	x
> 6 months after Tx	Individual	Individual	Individual

CMV, Cytomegalovirus; CRP, C-Reactive Proteine; EBV, Epstein-Barr Virus; LDH, Lactate dehydrogenase; PCR, Polymerase cghain reaction.

#### Other infection prophylaxis

Antifungal prophylaxis as a standard is not recommended. In protracted neutropenia, posaconazole, but also other drugs, such as fluconazole or itraconazole or micafungin, can be given according to local guidelines. Ofloxacin or ciprofloxacin for infection prophylaxis in neutropenia can be given until confirmed transplantation (engraftment) according to local guidelines. Pre-emptive CMV treatment with ganciclovir or valganciclovir can be administered if a replicative infection is proven (positive pp65 test or PCR).

#### Transfusions

All blood products for administration in the first year after transplantation must be irradiated. CMV-negative patients should be transfused only with CMV-negative blood products or with an appropriately adapted system. Platelet and red cell transfusions should be administered according to site guidelines and protocols for allogeneic stem cell transplantation.

#### Other therapeutics

Antiemetics are administered according to local practice procedures or institutional guidelines. Infusions are administered and managed according to local guidelines.

#### Haematological course monitoring

Monitoring for post-transplant lymphoproliferative disease (PTLD) is performed according to local practice.

All patients in aplasia should be treated in a single room, ideally with appropriate clean air facilities (laminar flow or HEPA) in accordance with the accreditation standards of the Joint Accreditation Committee ISCT-Europe and EBMT (JACIE).

#### Vaccinations after therapy

Vaccination protection for all vaccinations is usually lost through transplantation. Post-transplant vaccinations should be given according to the recommendations of the transplant societies with individual risk assessment in the MS context. Post-transplant vaccinations should be started 6 months after AHSCT with inactivated vaccines and 24 months after AHSCT with live attenuated vaccines, according to guidelines. In Germany, vaccine selection is based on availability and current Paul Ehrlich Institute (PEI) recommendations. According to EBMT guidelines, titre checks before vaccination are not recommended, and vaccinations can be carried out according to local standards.

From 3 months after transplantation:

- Influenza (tetravalent)
- Pneumococcus (Prevenar 13, then Pneumovax 23)
- VZV (Shingrix, inactivated vaccine)
- COVID-19 (SARS-CoV-2)

From 6 months after transplantation:

- Tetanus
- Diphtheria (full dose ‘D’ recommended)
- Pertussis (full dose, acellular vaccine ‘aP’ recommended)
- Poliomyelitis (use inactivated vaccine: IPV)
- Haemophilus influenzae type B (conjugate vaccine recommended)
- Meningococcal (meningococcal ACWY conjugate and meningococcal B vaccine)
- Hepatitis B (titre determination recommended after completion of vaccinations)

From 24 months after transplantation

- Measles/mumps/rubella (live vaccine) after titre control

#### Rehabilitation

MS-competent neurological rehabilitation from month 3 after transplantation should be sought for all patients, ideally in a clinic with expertise in AHSCT for MS or corresponding cooperation with a haemato-oncologist, as summarised in EBMT guidelines.^28^

#### Neuroimmunological follow-up

In the first year, follow-up should be quarterly, followed by half-yearly clinical controls. A cranial MRI and, ideally, a spinal MRI as ‘re-baseline MRI’ and safety assessment should be carried out 3–6 months after transplantation, then annually or earlier if clinically indicated. Although there is little evidence for any ongoing focal inflammation after AHSCT, the database is not sufficient to exclude evolution of new inflammatory activity after AHSCT.

### Study concept: German REgistry cohort of autoLogous haematopoietic stem cell transplantation in MS

German REgistry cohort of autoLogous haematopoietic stem cell transplantation in MS (RECLAIM) will combine two registry platforms, the German MS registry and the DRST, managed by DAG-HSZT, which is linked to the EBMT database. In addition, patients will be invited to contribute to the Observational Study in MS treated with aHSCT (OMST) of EBMT led by R. Saccardi. The aim is to document all pwMS living in Germany who have been treated with AHSCT, even if outside Germany. We will focus on the long-term effects of AHSCT on disability and MRI data, as well as safety, tolerability and toxicity, based on yearly clinical evaluations. To be included, patients are invited to approach 1 of the 160 local MS centres contributing to the MS registry. In addition, patients without access or unwilling to access an MS centre can provide their data to the central study centre in Hamburg and will be monitored remotely as well. The study start is planned for spring 2023.

## Declarations

**Ethics approval and consent to participate:** Ethics approval for RECLAIM has been given by 12 April 2023.

**Consent for publication:** Not applicable.

**Availability of data and materials:** Not applicable.
